# ZD6126 inhibits orthotopic growth and peritoneal carcinomatosis in a mouse model of human gastric cancer

**DOI:** 10.1038/sj.bjc.6601490

**Published:** 2004-02-03

**Authors:** M F McCarty, A Takeda, O Stoeltzing, W Liu, F Fan, N Reinmuth, M Akagi, C Bucana, P F Mansfield, A Ryan, L M Ellis

**Affiliations:** 1 Department of Surgical Oncology, The University of Texas MD Anderson Cancer Center, 1515 Holcombe Blvd., Box 444, Houston, TX 77030-4009, USA; 2 Department of Cancer Biology, The University of Texas MD Anderson Cancer Center, 1515 Holcombe Blvd., Box 444, Houston, TX 77030-4009, USA; 3 Cancer and Infection Bioscience Department, AstraZeneca, Macclesfield, UK

**Keywords:** endothelium, apoptosis, neovascularisation inhibitors, neoplasm metastasis

## Abstract

The purpose of this study was to examine the effects of ZD6126, a novel vascular-targeting agent, on tumour growth and angiogenesis in an orthotopic model of gastric cancer. TMK-1 human gastric adenocarcinoma cells were injected into the gastric wall of nude mice. After the tumours were established (day 14), therapy was initiated. Mice (*n*=11–12/group) received (a) vehicle, (b) ZD6126 at 100 mg kg day^−1^ i.p. one time per week or (c) ZD6126 at 100 mg kg day^−1^ i.p. five times per week. Tumour mass, volume and the presence or absence of peritoneal carcinomatosis were determined at sacrifice on day 38. Tumours from each group were stained for markers of blood vessels, proliferation and apoptosis. To further define the time frame of the vascular-targeting effects of chronic therapy with ZD6126, TMK-1 cells were again injected into the gastric wall of mice in a second experiment. On day 14, a single i.p. injection of ZD6126 100 mg kg^−1^ mouse^−1^ or vehicle was delivered. Groups of three mice each were killed and the tumours harvested at days 1, 3 and 5 post-ZD6126 injection. Tumours were processed and stained for endothelial and tumour cell apoptosis and proliferation. No overt toxicity was observed with ZD6126 therapy. ZD6126 led to a marked inhibition of tumour growth (82% decrease *vs* control (*P*<0.001)). ZD6126 also led to a significant decrease in the incidence of peritoneal carcinomatosis (10 out of 12 controls, *vs* one out of 12 ZD6126) (*P*<0.01). Histological analysis of tumours revealed large regions of central necrosis in the treated group, as well as a dramatic increase in tumour cell apoptosis (7.4-fold increase (*P*<0.001)), consistent with the vascular-targeting activity of ZD6126. Mice treated with ZD6126 demonstrated a 59% decrease in PCNA-positive cells (*P*< 0.02), indicating reduced tumour cell proliferation. In addition, tumours treated with ZD6126 exhibited a 40% decrease in microvessel density (*P*<0.05). Results from mice treated with a single injection of ZD6126 demonstrated the acute effects this agent has on the tumour vasculature. The ratio of endothelial cell apoptosis to endothelial cell proliferation was increased within 24 h of a single injection. In conclusion, ZD6126 significantly inhibited tumour growth and metastasis in an orthotopic model of human gastric adenocarcinoma, without detectable problematic adverse effects. These data suggest that ZD6126 may be worthy of investigation in the treatment of primary gastric adenocarcinoma.

The 5-year survival rate for patients with gastric cancer in the United States is only 5–15% (Karpeh *et al*, 2001). The mortality associated with gastric cancer has remained relatively stable over the past 20 years, suggesting that new therapies to combat this problem are urgently needed (Karpeh *et al*, 2001). Recent studies have shown that targeting the tumour vasculature may be of therapeutic benefit in gastric cancer (Mori *et al*, 2000; Yoshikawa *et al*, 2000; Jung *et al*, 2002; Kamiyama *et al*, 2002).

The generation and maintenance of a functional vasculature is necessary for tumours to increase in size beyond 1–2 mm (Folkman, 1992). Anti-angiogenic agents target biological processes involved in the generation of new blood vessels, for example, by blocking the receptor-ligand systems important for the angiogenic response (Bruns *et al*, 2000; Jung *et al*, 2002). However, by the time the cancer is diagnosed, most tumours already have an intact, functional vascular network (Folkman, 1992). Significantly, tumour vasculature seems to be fundamentally different from normal vasculature (Denekamp, 1990). For example, tumour endothelium is characterised by tortuous, leaky vessels with chaotic blood flow (Fukumura *et al*, 1997) and a significant proportion of immature, proliferating endothelial cells, which is in contrast to the ordered vessels and quiescent endothelial cells found in normal tissues (Skinner *et al*, 1995; Brown and Giaccia, 1998). Targeting the phenotypic differences between tumour vasculature and normal vasculature is particularly attractive because it has the potential to be effective against established tumours, which are often considered to be resistant to standard chemotherapeutic approaches.

In contrast to anti-angiogenic therapies, vascular targeting therapies aim to selectively and irreversibly disrupt tumour blood flow, inducing prolonged ischaemia and subsequent secondary necrotic tumour cell death (Reviewed in (Chaplin and Hill, 2002). A wide range of potentially effective vascular-targeting approaches has been described, each exploiting distinctive features of the tumour vasculature (Denekamp, 1990; Bloemendal *et al*, 1999; Thorpe *et al*, 2003). Vascular-targeting activity is a common feature of tubulin-binding microtubule destabilising agents such as colchicines and vinca alkaloids. Although these agents have significant vascular-targeting activity in animal tumour models (Chaplin *et al*, 1996) this is only seen at or around their maximum tolerated dose (MTD). More than half a century ago, colchicine, one of the oldest known tubulin-binding agents, was found to cause extensive central necrosis and hemorrhage of tumours when administered to cancer patients, presumably through vascular-targeting activity (Seed *et al*, 1940). However significant toxicity prevented its further clinical development as an anti-cancer agent. Therefore, newer agents that more selectively disrupt tumour vasculature with a wider therapeutic margin over normal tissue effects have been sought for clinical evaluation. In particular, two compounds currently in phase II clinical development (combretastatin A-4 phosphate and ZD6126) have demonstrated selective effects on tumour vasculature in animal models at doses significantly below their MTD (Dark *et al*, 1997; Davis *et al*, 2002). Although the molecular mechanisms underlying the vascular-targeting activity of these agents is not well understood, a range of *in vitro* endothelial cell responses (eg cytoskeletal reorganisation, cell shape change) and *in vivo* vascular responses (eg vascular collapse, increased permeability, decreased perfusion) have been described that could play a significant role in their anti-tumour effects (Griggs *et al*, 2001; Blakey *et al*, 2002; Davis *et al*, 2002).

ZD6126, is a novel vascular-targeting agent that was developed for its ability to bind tubulin and induce vascular damage in tumours (Davis *et al*, 2002). It has a wide therapeutic index in mouse tumour models producing selective antivascular effects in tumours at doses ranging from 1/8th to 1/16th of the MTD in mice (Davis *et al*, 2000; Blakey *et al*, 2002; Siemann and Rojiani, 2002). ZD6126 has significant antitumour effects in a wide range of histologically distinct subcutaneous tumour xenografts in nude mice (Blakey *et al*, 2002). However, the efficacy of ZD6126 has not been examined at orthotopic sites where the endothelia are phenotypically distinct and tumour growth is likely to be more physiologically relevant to human disease (Craig *et al*, 1998; Chi *et al*, 2003).

The aim of this study was to determine the efficacy of single-agent ZD6126 therapy on tumour growth and vasculature in an orthotopic model of gastric cancer.

## MATERIALS AND METHODS

### Cell culture

TMK-1, a poorly differentiated human gastric adenocarcinoma cell line, was a generous gift of Dr Eiichi Tahara (Hiroshima University, Hiroshima, Japan), and was cultured and maintained in Dulbecco's modified Eagle medium supplemented with 10% fetal bovine serum, as described previously (Jung *et al*, 2002).

### Formulation of ZD6126

ZD6126, a phosphate prodrug of *N*-acetylcolchinol, was provided by AstraZeneca (Macclesfield, England). ZD6126 was dissolved in 5% sodium carbonate and diluted to the final volume with phosphate-buffered saline (PBS), as previously described (Blakey *et al*, 2002). Solutions were sterilised by filtration through a 0.22-*μ*m filter and stored at 4°C.

### Animal studies

Male athymic mice, 8-week old (obtained from the National Cancer Institute Animal Production Area, Frederick, MD, USA), were acclimated for 1 week and caged in groups of five. All animal studies were conducted under the guidelines approved by the Animal Care and Use Committee of The University of Texas MD Anderson Cancer Center, and met all of the standards required by the UKCCCR guidelines for the welfare of animals in experimental neoplasia, as published (Workman *et al*, 1998).

**Figure 1:**
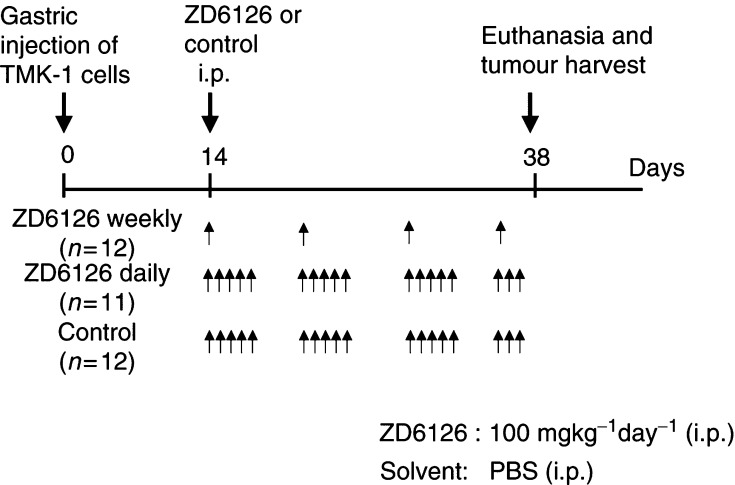
Experimental design of ZD6126 therapy for gastric cancer growing in an orthotopic site. TMK-1 human gastric cancer cells (10^6^) were injected into the gastric walls of nude mice. Mice were randomised to one of three groups: control, ZD6126 1 day week^−1^, or ZD6126 5 days week^−1^. Treatment with ZD6126 (100 mg kg^−1^) or control began on day 14 by i.p. injection. On day 38, all mice were euthanised.

Mice were anaesthetised by i.p. injection of sodium phenobarbital (Nembutal, 50 mg kg^−1^) and, under sterile conditions, subjected to an upper midline laparotomy. TMK-1 cells (10^6^) in Hank's balanced salt solution were injected into the wall of the mid-stomach. After 14 days, when tumours were approximately 1–2 mm in diameter, mice were randomly assigned to one of three groups: (a) daily-dose control (PBS+5% sodium carbonate, pH 7.0, *n*=12); (b) weekly-dose ZD6126 (100 mg kg^−1^, 1 day week^−1^); and (c) daily-dose ZD6126 (100 mg kg^−1^, 5 days week^−1^, *n*=11) (Figure 1

). The weekly dose group received four injections of ZD6126 on days 14, 21, 28 and 35, and received the last injection 3 days prior to sacrifice. The daily dose group received 18 injections (days 14–18, 21–25, 28–32, 35–37) and received the last injection 24 h prior to sacrifice. Body weight at randomisation was similar among groups and animals in all groups gained weight at a similar rate throughout the experiment. Since this tumour model can result in animals rapidly becoming moribund from bowel obstruction, mice were very closely monitored for signs of loss of clinical condition associated with morbidity. In this experiment, all mice were euthanised by CO_2_ asphyxiation on day 38 when 25% of the control mice showed clinical signs (lack of activity and increase tumour burden on palpation), indicating that they would be expected shortly to become moribund. Body weights were measured, tumours were excised, tumour weight and diameters were subsequently determined and the presence or absence of peritoneal carcinomatosis was noted. Tumour volumes were calculated by the equation width^2^ × length × 0.5. Tumour tissue was harvested and placed in either 10% buffered formalin for paraffin fixation or optimal cutting temperature (OCT) compound (Miles Inc., Elkhart, IN, USA) and frozen in liquid nitrogen for subsequent immunohistochemical analysis.

In a separate experiment to clarify the timing of the antivascular effect of ZD6126, mice bearing established orthotopic tumours were injected with a single dose of ZD6126 (100 mg kg^−1^) or control solution (PBS+5% sodium carbonate) 14 days after tumour-cell implantation. These mice were injected i.p. with 1 mg/0.2 ml of 5-bromo-2′-deoxyuridine (BrdU) (Sigma, St Louis, MO, USA) dissolved in PBS 1 h before euthanasia to determine the fraction of proliferating cells. Three mice per group were euthanised at days 1, 3, or 5 after a single injection of ZD6126, and the tumour tissues were harvested as above.

### Immunostaining of microvessels, proliferative and apoptotic cells

Tissue sections were sectioned and stained for H&E, CD31 (vessels), TUNEL (apoptotic cells), BrdU (proliferative cells) and PCNA (proliferative cells), as previously described (Reinmuth *et al*, 2002; Stoeltzing *et al*, 2003). All tumour tissues were counterstained either with Gill's 3 haematoxylin (Sigma; immunochistochemical analysis), or incubated with 300 *μ*g/ml of Hoechst stain for 1–2 min (Sigma, immunofluorescent analysis). The antibodies used were as follows: rat anti-mouse CD31 (Pharmingen, San Diego, CA, USA), mouse anti-BrdU (Becton Dickinson, Franklin Lakes, NJ, USA), mouse anti-human PCNA PC-10 (DAKO, Carpinteria, CA, USA), DeadEnd Fluorometric TUNEL system (Promega, Madison, WI, USA), rat anti-mouse IgG_2a_ (Serotec, Raleigh, NC, USA), Texas Red goat anti-rat secondary antibody (Jackson Research Laboratories, West Grove, CA, USA) and goat anti-mouse IgG Alexa Fluor 488 (Molecular Probes, Eugene, OR, USA).

### Analysis of immunostained tissue sections

Sections were examined using a Zeiss photomicroscope (Carl Zeiss Inc., Thornwood, NY, USA) equipped with a 3-chip charge-coupled device colour camera (DXC-960 MD; Sony Corp., Tokyo, Japan). The images were analysed using Optimas image analysis software (version 5.2; Bothell, WA, USA). Positive cells were counted using Scion software, based on the NIH Image program for Macintosh (Scion Corporation, Frederick, MD, USA). Quantification of positive cells was expressed as the average of the number of cells in 0.05-mm^2^ high-power fields at × 200. Five fields from one 8–10 *μ*m section per tumour specimen were chosen randomly and 3–5 specimens per group underwent analysis. Each field contained more than 2000 cells. Areas of obvious necrosis (as determined by either the haematoxylin or Hoechst counterstain) were avoided. The ratio of endothelial cell apoptosis to endothelial cell proliferation was determined by counting the number of CD31-positive/TUNEL-positive cells divided by the number of CD31-positive/BrdU-positive cells per high-power field.

### Statistical analysis

Statistical differences among groups were examined using the two-tailed Student's *t*-test, the *χ*^2^ test, or, for analysis of nonparametric data, the Mann–Whitney *U*-test with InStat Statistical Software (GraphPad Software, San Diego, CA, USA). The results of the *in vivo* experiments were tested for outliers using Grubb's test (www.graphpad.com). A *P*-value of less than 0.05 was considered statistically significant.

## RESULTS

### Effect of ZD6126 on growth of primary gastric tumours

No difference was discerned in the body weight or grooming habits of the treatment groups (multiple-dose ZD6126, single-dose ZD6126, or control). The effects of ZD6126 on the growth of TMK-1 cells implanted into the stomach walls of nude mice are shown in Table 1

**Table 1:** Treatment of gastric cancer with ZD6126

		**ZD6126** (**100 mg kg**^−1^)	
	**Control**	**Weekly**	**Daily**
Body mass (g)±s.e.m.	30.9±0.8	31.3±1.0	29.2±0.7
Tumour volume (mm^3^)±s.e.m.	440±154	241±39^b^	79±25^a^
Tumour mass (g)±s.e.m.	0.5±0.1	0.4±0.1	0.2±0.1^b^
Incidence of carcinomatosis	10/12 (83.3%)	6/12 (50%)	1/11 (9.1%)^c^

TMK-1 was injected into the gastric wall on day 0. Therapy with ZD6126, either once a week (weekly) or 5 × per week (daily), was initiated on day 14. Mice were killed on day 38.

a *P*<0.002 by Student's *t*-test.

b *P*<0.05 by Student's *t*-test.

c *P*<0.01 by *χ*^2^ analysis.

**Figure 2:**
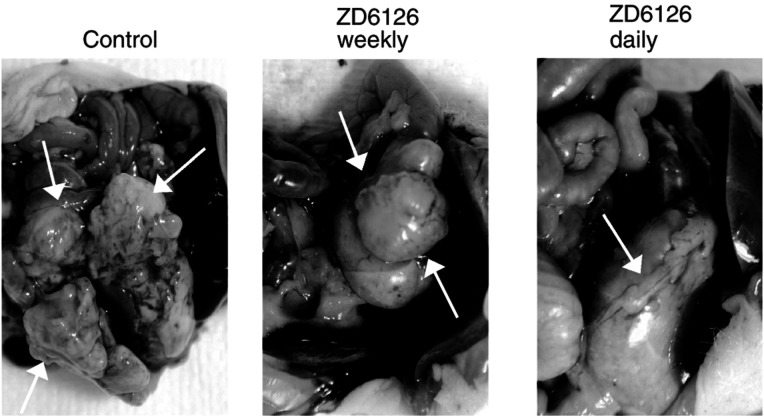
Effects of ZD6126 on gastric tumour growth. TMK-1 cells (10^6^) were injected into the stomach walls of nude mice, and therapy was begun on day 14. Photographs show that daily ZD6126 administration was more effective in inhibiting tumour growth than daily administration or control.

. Tumour volume was reduced in both the weekly-dose group (45% decrease, *P*< 0.05) and the daily-dose group (82% decrease, *P*<0.002) (Figure 2

, Table 1). Tumour mass was less in the group given daily ZD6126 than in the control mice (*P*<0.05), but weekly ZD6126 did not significantly reduce tumour mass relative to that in the control group (*P*=0.26).

### Effect of ZD6126 on incidence of peritoneal carcinomatosis

TMK-1 cells are known to induce peritoneal carcinomatosis in a similar mouse model (Minagawa *et al*, 2001). In our study, daily ZD6126 therapy led to a reduction in the incidence of peritoneal deposits: 83% of the control mice developed gross peritoneal carcinomatosis, and this was reduced to less than 10% of mice treated with daily ZD6126 (*P*<0.01) (Table 1). Weekly ZD6126 did not significantly affect the development of carcinomatosis (50% incidence, *P*=0.08 *vs* controls).

### Decreased microvessel density in ZD6126-treated animals

Immunohistochemical staining for CD31 to determine the microvessel density revealed no difference between the control mice and those given weekly ZD6126. However, microvessel density was reduced by 40% in mice treated daily with ZD6126 relative to that in the control group (*P*<0.05) (Table 2

**Table 2:** Treatment of gastric cancer with ZD6126

		**ZD6126 (100 mg kg^−1^)**	
	**Control**	**Weekly**	**Daily**
Microvessel density # of vessels/HPF	12.0±1.5	10.1±1.2	7.2±1.6^a^
Proliferating cells # of cells/HPF	16.9±2.5	8.0±1.1^b^	7.0±1.3^a^>
Apoptotic cells # of cells/HPF	2.0±0.4	10.8±3.7^b^	15.0±3.0^c^

TMK-1 was injected into the gastric wall on day 0. Therapy with ZD6126, either once a week (weekly) or 5 × per week (daily), was initiated on day 14. Mice were killed on day 38. Tumours were harvested and tissue sections were stained with antibodies against CD31 to determine the microvessel density, PCNA to determine the cycling cells, or TUNEL to determine apoptotic cells. Measurements are the mean±the standard error, as determined by counting the positive cells in each of five × 200 high-powered fields (HPF) for at least five tumours from each treatment or control group.

a *P*<0.05.

b *P*<0.01.

c *P*<0.001.

, Figure 3

**Figure 3:**
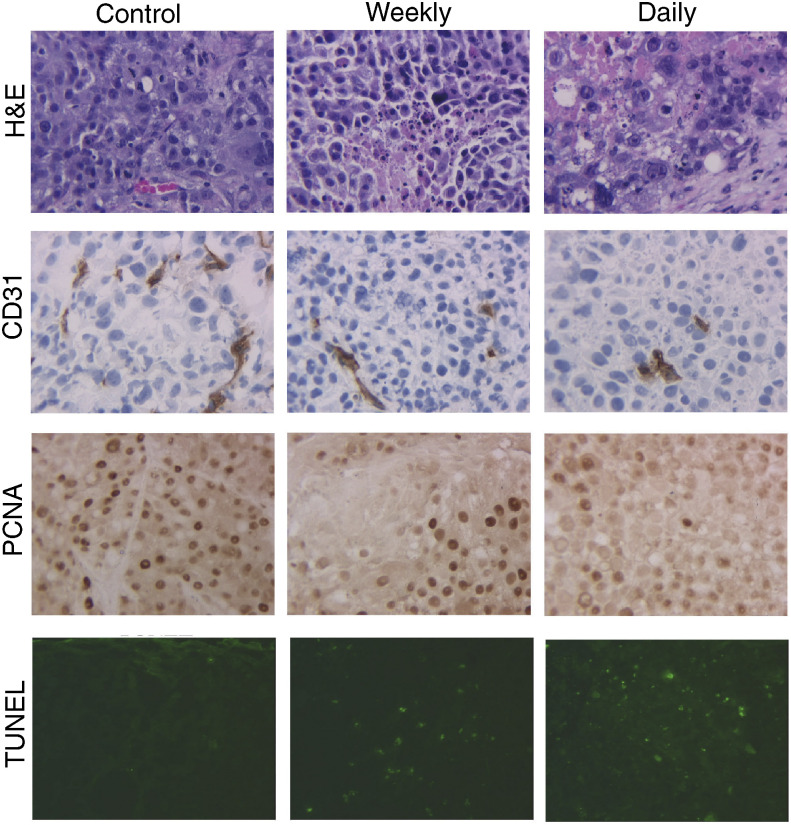
Immunohistochemical analysis of gastric tumours. Representative images of tumour sections stained with haematoxylin and eosin (row 1; × 200 magnification), stained immunohistochemically for CD31 (row 2; × 200) or for PCNA (row 3; × 200), or stained for apoptosis by TUNEL (row 4, × 200). Weekly ZD6126 caused a significant decrease in the number of PCNA+ proliferating cells and an increase in TUNEL-positive apoptotic cells; daily ZD6126 decreased microvessel density (as shown by staining with antibodies against CD31), decreased proliferating cells (PCNA) and increased apoptotic cells (TUNEL).

).

### Tumour cell proliferation and cell death in ZD6126-treated animals

The proliferative and apoptotic rates of the tumour cells in each of the three groups are shown in Table 2 and Figure 3. Significantly fewer PCNA-positive cells were found in tumours in the ZD6126-treated groups (53% fewer in the weekly group (*P*<0.01) and 58% fewer in the daily group (*P*<0.05)), concomitant with significant increases in TUNEL-positive cells in both groups (5.3-fold increase in the weekly group (*P*<0.007) and 7.4-fold increase in the daily group (*P*<0.001)).

### Endothelial cell apoptosis and proliferation

Tumours harvested from mice treated with ZD6126 either weekly or daily for 38 days were stained for markers of endothelial-cell apoptosis (CD31/TUNEL), to determine whether the decrease in microvessel density was associated with increased endothelial cell apoptosis. However, at that relatively late time point, no apoptotic endothelial cells were detected (data not shown). Therefore, to examine the acute effects of ZD6126 on the tumour microvasculature, mice bearing established tumours were injected with a single dose of ZD6126 (100 mg/kg) on day 14, and tumours were harvested 1, 3, or 5 days after the injection. As shown in Table 3

**Table 3:** Treatment of gastric cancer with ZD6126: time course

		**ZD6126 (100 mg kg^−1^)**		
	**Control**	**Day 1**	**Day 3**	**Day 5**
Tumour weight (g)	0.6±0.3	0.3±0.06	0.2±0.01	0.3±0.02
Tumour volume (mm^3^)	250.1±75.7	242.6±31.0	167.8±47.3	134.2±34.6
Proliferating cells # of cells/HPF	112.3±7.3	43.7±14.7^a^	78.4±14.6^b^	33.0±10.0^a^
Apoptotic cells # of cells/HPF	0.8±0.3	12.0±6.6^b^	16.2±7.9^c^	7.2±3.3^b^
Microvessel density # of vessels/HPF	32.9±2.7	29.3±2.4	25.7±2.9	22.8±2.6^a^
Proliferating EC # of cells/HPF	2.5±0.4	1.1±0.3^b^	1.1±0.18^c^	1.3±0.3^b^
Apoptotic EC # of cells/HPF	0.6±0.1	0.7±0.3	1.2±0.3	0.6±0.1
Ratio of apoptotic to proliferating ECs (% control)	0.3	0.6^c^ (57%)	1.1^c^ (67%)	0.8^b^ (68%)

TMK-1 tumours growing in the gastric wall were harvested at 1 day, 3 days and 5 days post-ZD6126 injection. Tissue sections were stained with antibodies against CD31, BrdU and TUNEL to determine MVD, endothelial cell proliferation and endothelial cell apoptosis. Measurements are the mean±the standard error, as determined by counting the positive cells in each of five × 100 high-powered fields (HPF) for three tumours from each treatment or control group. EC=endothelial cell.

a *P*<0.004.

b *P*<0.05.

c *P*<0.01.

, at day 1 post-ZD6126 injection, there was a significant decrease in the number of proliferating cells, as determined by the percent of BrdU-positive cells (61% decrease (*P*<0.004)). There was also a concomitant increase in cell death at day 1, which was sustained through to day 5 (15-fold increase at day 1 (*P*<0.05) and nine-fold increase at day 5 (*P*<0.05)). Importantly, there was also a significant decrease in endothelial cell proliferation by day 1 post-ZD6126 injection (56% decrease in CD31+/BrdU+ cells (*P*<0.05)). However, since the process of angiogenesis is determined by a balance between proliferating and apoptotic endothelial cells, the ratio of these two parameters was determined. As shown in Table 3, ZD6126 led to a significant increase in the ratio of endothelial cell apoptosis to proliferation (57%: *P*<0.01). This increase was apparent as early as 24 h post-ZD6126 injection, and continued through day 5.

## DISCUSSION

Our findings in this study demonstrate the efficacy of selectively targeting the tumour vasculature with the novel antivascular agent ZD6126 in an orthotopic model of human gastric cancer. Previous studies have described ZD6126, both as a single agent and in combination with radiation and chemotherapy, in various models in which tumour cells are implanted in subcutaneous tissues (Blakey *et al*, 2002; Siemann *et al*, 2002; Siemann & Rojiani, 2002). Here we focus on the efficacy of single-agent ZD6126 therapy, administered in two dose schedules, in an orthotopic model of gastric cancer.

Daily dosing with ZD6126 (100 mg kg^−1^, 5 days per week) caused a significant inhibition of tumour growth. Once-weekly dosing, in contrast, reduced the tumour volume, but did not significantly reduce tumour mass. This difference may be due to the variability of excising and weighing the tumour, as it is difficult to ensure complete removal of the tumour, without the accompanying uninvolved stomach wall. This is reflected by the proportionately greater variability in the tumour mass measurement, especially as the tumour volume is reduced. Previous studies in subcutaneous xenograft tumour models have shown that a single bolus dose of ZD6126 produces widespread central tumour necrosis with a rim of viable tumour cells surviving treatment, which can quickly repopulate the tumour mass, resulting in limited tumour growth delay (Blakey *et al*, 2002). These tumour cells are not killed following drug treatment, presumably because they gain oxygen and nutrients from blood vessels in the surrounding normal tissue, which is unaffected by ZD6126 (Blakey *et al*, 2002). Therefore, in a rapidly growing tumour model system, repeated dosing of ZD6126 is required to obtain significant tumour growth delays (Blakey *et al*, 2002; Davis *et al*, 2002). We observe greater antitumour effects with the daily dosing schedule, which may reflect a greater opportunity for regrowth of tumour between doses of ZD6126 in the weekly schedule.

Examination of the tumour samples at the end of the experiment (day 38), 24 h after the last dose of ZD6126 was administered, showed that both treatment schedules produced significant antitumour effects in terms of increased tumour cell death (TUNEL-positive staining), together with decreased tumour cell proliferation (PCNA-positive staining). However, the TUNEL-staining procedure can detect both apoptotic and necrotic cell death (Grasl-Kraupp *et al*, 1995), and, therefore, it does not clearly discriminate between these two modes of cell death. Despite that, the increase in TUNEL-positive cells is more likely due to an increase in apoptosis as areas of intense necrosis (as determined either by H&E staining or the Hoechst counterstain) were avoided. Additionally, many of the fields that were chosen had only diffuse TUNEL-positive cells surrounded by areas of viable tissues, again indicative of apoptosis and not necrosis. Tumours isolated from animals following weekly or daily dosing schedules of ZD6126 appeared to have a higher degree of morphological tumour necrosis on H&E sections (data not shown). Taken together with the increase in TUNEL staining in the tumours compared with the controls, this would be consistent with a vascular-targeting mechanism of action for ZD6126 in this anti-tumour study.

In these experiments, we did not observe increased endothelial cell death in gastric tumours examined at the end of either the daily or weekly ZD6126 treatment schedule. However, ZD6126 treatment has been shown to induce rapid occlusion of tumour blood vessels (within 6 h), probably as a result of drug-induced effects on endothelial cell shape, leading to exposure of basement membrane and rapid induction of endothelial cell apoptosis and subsequent loss of vessel function (Blakey *et al*, 2002). Therefore, detecting endothelial cells undergoing apoptosis after multiple rounds of treatment may be very difficult, since the bulk of the potentially ZD6126-sensitive endothelial cells throughout the tumour would be affected at the earliest times of the treatment regimen. At later times, endothelial cell proliferation and vessel growth would be expected to be more localised around areas of the tumour that are nourished from nontumour vessels (e.g. at the tumour rim) which are unaffected by ZD6126 (Blakey *et al*, 2002).

**Figure 4:**
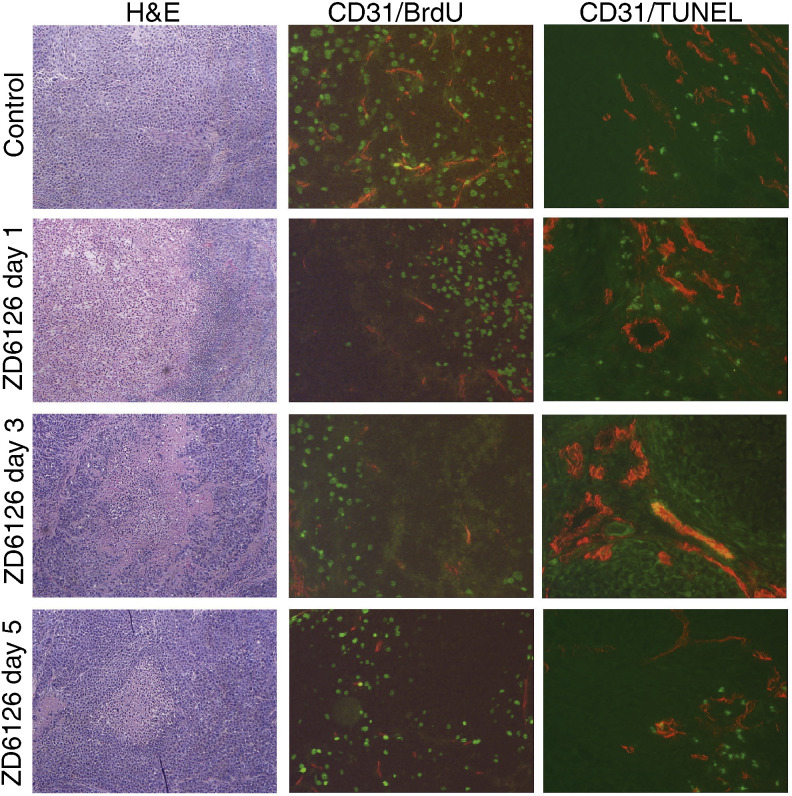
Immunohistochemical analysis of gastric tumours after a single dose of ZD6126. Representative images of tumour sections stained with haematoxylin and eosin (column 1; × 40 magnification), stained for endothelial cell proliferation by immunofluorescence for CD31/BrdU (column 2; × 100) and stained for endothelial cell apoptosis by immunofluorescence against CD31/TUNEL (column 4, × 100). A single dose of ZD6126 caused a significant decrease in the number of dividing endothelial cells (as determined by colocalisation) and an increase in endothelial cells undergoing apoptosis, such that microvessel density was decreased by day 5.

To further investigate the dynamics of ZD6126 effects on endothelial cell apoptosis and proliferation, we treated mice bearing established tumours with a single injection of ZD6126, and harvested the tumours 1, 3 and 5 days later. Immunohistochemical analysis revealed that microvessel density tended to decrease following ZD6126 injection, reaching statistical significance on day 5. This corresponded with a decrease in proliferating endothelial cells following ZD6126 treatment. Taken together, these observations may indicate that TMK-1 tumour regrowth and revascularisation are decoupled at early times after ZD6126 treatment, perhaps because angiogenesis is not rate limiting to tumour growth in the early-stage gastric tumours. Examination of H&E sections revealed that widespread necrosis was evident 1–3 days after ZD6126 treatment. Multiple areas of focal necrosis were evident throughout the tumour mass, surrounded by regions of viable tumour tissue. By day 5, the extent of histological tumour necrosis was reduced, as demonstrated in Figure 4

. This most likely represents tumour regrowth, as has been described in rapidly proliferating subcutaneous xenograft models (Blakey *et al*, 2002).

Previously, ZD6126 was shown to have significant antitumour activity in a human lung pulmonary metastasis model in nude mice (Goto *et al*, 2002). In this metastasis model, ZD6126 antitumour activity was associated with selective induction of apoptosis in tumour endothelial cells 24 h after ZD6126 treatment, with associated destruction of the tumour vasculature. However, despite the widespread tumour necrosis in the lung metastases, repeated dosing was required to reduce disease burden, consistent with what has been described for other rapidly growing tumours (Blakey *et al*, 2002; Davis *et al*, 2002). In the present study, ZD6126 treatment reduced the incidence of peritoneal carcinomatosis, which may be a consequence of reducing the growth of the primary gastric tumour rather than a direct effect on the metastatic deposits in the peritoneum. In contrast to the study by Goto *et al* (2002), we did not see a significant increase on endothelial cell apoptosis within the tumour even at 24 h after ZD6126 treatment. The reason for the difference between the two studies is not certain, but it is possible that the apoptotic response of tumour endothelial cells to vascular-targeting agents may depend on the tumour microenvironment within each disease site. However, despite the differences in induction of tumour endothelial cell apoptosis, ZD6126 produced significant antitumour effects in both disease models.

As reported by others (Blakey *et al*, 2002; Goto *et al*, 2002), the effects of ZD6126 appear to be very selective for tumour vasculature. We did not observe any increase in cell death within the histologically normal tissues surrounding the tumour, either in epithelial or endothelial cells (data not shown).

The activity of combretastatin A-4 phosphate has been reported in several physiologically relevant mouse models, including orthotopic models of mouse (MAC 15) or human (SW620) colorectal cancer (Grosios *et al*, 1999), models of human colon cancer (DLD-1 and HT29) metastasis to the liver (Holwell *et al*, 2002) and orthotopic models of human NSCLC (KNS-62, Colo-699) (Boehle *et al*, 2001). In the first of these studies, a single dose of combretastatin A-4 phosphate (100 mg/kg) induced extensive haemorrhagic necrosis, with a rim of viable tumour at the periphery of the primary colorectal tumour 24 h after drug treatment. This effect was also observed in vascular metastatic deposits in the kidney, lymph nodes and abdominal wall, but no effect was observed in avascular deposits in the lung (Grosios *et al*, 1999), supporting the hypothesis that the tumour necrosis induced by the drug is secondary to vascular effects in the orthotopic setting. In the follow-up study, a single dose of combretastatin A-4 phosphate (150 mg/kg) also induced haemorrhagic necrosis in the colorectal tumours within the liver 24 h after drug treatment (Holwell *et al*, 2002). The extent of necrosis induction was significantly lower when SW620 was grown in the liver rather than as a subcutaneous tumour, indicating that the tumour microenvironment can affect the response of a tumour to vascular-targeting agents. In the final study of two human NSCLC xenografts, a daily regimen of combretastatin A-4 phosphate (50 mg kg^−1^ day^−1^ for 21 days) significantly inhibited tumour growth in both the subcutaneous and orthotopic settings (Boehle *et al*, 2001).

The present study has shown that the vascular-targeting agent ZD6126 was effective in reducing the growth and peritoneal spread of human gastric cancer cells implanted orthotopically into the stomach walls of nude mice. Furthermore, the antitumour activity was improved with daily dosing compared with weekly dosing. Further studies are needed to more completely define the mechanism of action of this agent and to determine whether additional antitumour effects can be seen in this model in combination with clinically relevant chemotherapy, radiation therapy, or antiangiogenic agents. However, the present preclinical results provide support for investigating the potential activity of ZD6126 in gastric cancer in the clinic.
